# Mind the level: problems with two recent nation-level analyses in psychology

**DOI:** 10.3389/fpsyg.2014.01110

**Published:** 2014-09-30

**Authors:** Toon Kuppens, Thomas V. Pollet

**Affiliations:** ^1^Faculty of Behavioural and Social Sciences, University of GroningenGroningen, Netherlands; ^2^Faculty of Psychology and Education, VU University AmsterdamAmsterdam, Netherlands

**Keywords:** statistical analysis, cross-cultural differences, ecological fallacy, environmental performance, country age, wealth, religious beliefs, meaning in life

## Introduction

Two recent articles (both published in *Psychological Science*) rely on nation-level data to address questions about psychological processes: Oishi and Diener (2014), referred to here as “OD,” and Hershfield et al. (2014), referred to as “HBW.” In our opinion, both articles contain problems with regard to the use and interpretation of nation-level data. The problems are (1) the failure to account for the statistical dependence of countries within regions, (2) the use of nation-level data with questionable reliability, and (3) a confusion between individual and national levels of analysis.

## Galton's problem: neighboring countries tend to be similar

**Figure 1:**
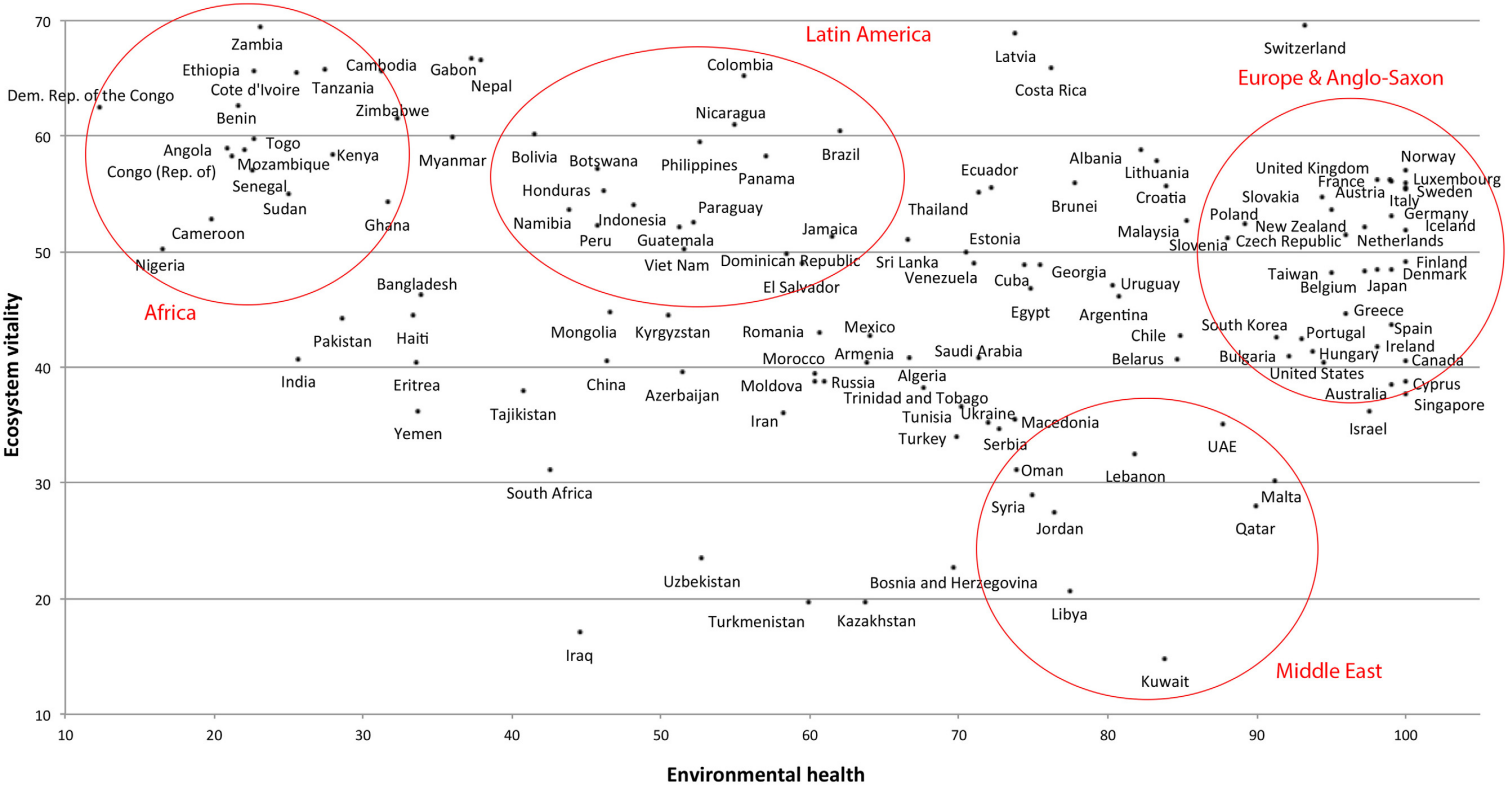
**A scatterplot of Environmental Health and Ecosystem Vitality shows that the scores are clustered by geographical region (circles indicate a high concentration of countries from a particular region)**.

First, the country-level analyses presented by OD and HBW violate the assumption of independent sampling (i.e., the statistical assumption of independence of errors) as countries are geographically (and historically) clustered (Ross and Homer, 1976; Pollet et al., 2014). This is also known as “Galton's problem.” Countries from the same region have undergone a similar development and cannot be considered independent data points. As a consequence, all significance tests are biased (Kruskal, 1988). This applies to OD, HBW, and many other nation-level analyses. We illustrate the consequences of Galton's problem with data from HBW. HBW presented results showing that country age was positively related with the Environmental Performance Index (EPI). The EPI consists of the subscales Environmental Health (EH) and Ecosystem Vitality (EV); Figure 1 clearly shows that the scores cluster by region.

The (unstandardized) effect of country age on EPI reported by HBW is 0.039, in a model controlling for GDP and the World Governance Indicators (and we replicate this in our own analysis). What happens when one takes into account the regional clustering of the data? Adding region (see Supplementary Material) to the model as a series of dummy variables reduces the coefficient to 0.018 (a reduction of 54%) and increases its standard error from 0.011 to 0.017, which means that the effect of country age is no longer significant (*p* = 0.12). Please note that our particular division of countries into regions is merely based on our own assessment of geographical proximity and cultural and political similarities. Our “region” variable could therefore easily be criticized or amended in many ways. As a robustness check, we have also used a different division into 10 rather than 12 regions that are more in line with Murdock's world regions (see Murdock and White, 1969, and http://en.wikipedia.org/wiki/Standard_cross-cultural_sample). We also repeated the analysis using 19 “UN geographical regions for statistical use” (see http://unstats.un.org/unsd/methods/m49/m49regin.htm). Both results were very similar to the results reported here. Finally, our results also hold when region is added as a random rather than a fixed effect.

## Reliability of nation-level data

The second problem is the use of nation-level data with poor reliability. OD compare suicide rates from around the world. This is problematic given that suicide registration and its underreporting varies substantially between countries, leading to a systematic bias (a lack of measurement equivalence, see Poortinga, 1989). There is considerable variation in this bias even for countries within the same region (Reynders et al., 2011).

While the measurement equivalence of suicide rates is debatable, the composition of HBW's main dependent variable is undeniably problematic. The EPI that HBW use as a dependent variable consists of two *negatively* correlated subscales. In other words, both subscales were intended to measure an aspect of environmental performance, but they correlate *negatively* (*r*_(132)_ = −0.26). Countries performing well on the first subscale are likely to perform poorly on the second subscale, and vice versa. Such a relation is of course opposite to what would be expected if the two subscales formed a reliable index of environmental performance. It is therefore better to analyze them separately rather than taking their (weighted) average. We have done so with the HBW data. Performing the analyses separately for EH and EV shows that country age is significantly related to EV (*B* = 0.052, *SE* = 0.018, *p* = 0.004), but not to EH (*B* = 0.008, *SE* = 0.025, *p* = 0.74). When additionally controlling for region, the effect of country age on EV was nearly halved and not significant anymore (*B* = 0.028, *SE* = 0.023, *p* = 0.22) and there still was no support for a relationship between country age and EH (*B* = −0.006, *SE* = 0.027, *p* = 0.82). Thus, the relationship between country age and the EPI or its subscales seems to be much smaller when one analyses the subscales separately and controls for the regional clustering of scores.

## Ecological fallacy: individual processes cannot be inferred from nation-level data

Third, the conceptual problem with both articles is that they use nation-level data to draw inferences at the individual-level. Both articles almost exclusively cite theories at the individual level, but empirical tests mainly rely on nation-level data. Both articles therefore contain an *ecological fallacy* (Robinson, 1950; Piantadosi et al., 1988). A correspondence between the level of theory and the level of analyses is important because relationships between variables can be fundamentally different at different levels, both theoretically and empirically. In other words, an individual-level relation can have the opposite sign of a nation-level relation. We illustrate the *ecological fallacy* with data from OD. OD report a correlation of −0.50 between religiosity and life satisfaction, but at the individual-level, meta-analyses show the *opposite* effect (Hackney and Sanders, 2003; Smith et al., 2003). OD report a positive correlation of 0.28 between national wealth and suicide rate at the national level, but there is a near-universal *negative* correlation at the neighborhood level (Rehkopf and Buka, 2006). Similarly, at the individual-level *low* (rather than high) socioeconomic status is a risk factor for suicide (Kessler et al., 1999; Nock et al., 2008; Borges et al., 2010) and causes depression (Ritsher et al., 2001; Wang et al., 2010). Finally, the reported non-significant nation-level correlation (*r* = 0.02) between life satisfaction and suicide is clearly positive at the individual-level (Koivumaa-Honkanen et al., 2001; Valois et al., 2004).

HBW partly deal with the *ecological fallacy* by presenting an individual-level experiment as a second study (even though participants were from a single country, limiting generalizability). OD actually have access to both individual-level and nation-level data. Yet, they do not present a multilevel model separating one level from the other. OD report relations between national wealth, religiosity, and meaning in life, but they do not control for individual wealth or take other individual-level variables into account together with nation-level variables. Crucially, their main finding is a negative correlation between national wealth and meaning in life. In an additional analysis, we therefore controlled for individual income (i.e., in addition to national wealth). Income was a 29-category variable ranging from 0 = $0, 1 = less than $1 a day, 2 = $1 to less than $2 a day to 28 = $125,000 a year or more. All local currencies were recoded into U.S. dollar and the variable was used as a continuous variable in the analyses. Thus, individual income is comparable across nations and can to some extent be seen as the individual-level equivalent of GDP per capita (which is OD's measure of national wealth). This analysis shows that individual income had a *positive* rather than a negative relationship with meaning in life, *B* = 0.004, *SE* < 0.001, *p* < 0.001, in a model that also controls for sex, age, marital status, individual religiosity, country religiosity, and GDP per capita. Thus, wealth seems to have *opposite* effects at the individual-level than at the country level. Given that OD discuss mainly individual-level processes in their Introduction and Discussion sections (they draw primarily on Baumeister, 1991), it seems that the individual-level relationship is a more appropriate test of their hypothesis than the nation-level relationship. If one agrees that individual-level theories should be tested with individual-level data, the results are opposite to OD's predictions. The title of the OD article is correct in that GDP per capita correlates *negatively* with the country average of meaning in life. However, none of the theories or mechanisms discussed in the paper offer an understanding of this relation because they are at the individual rather than the country level.

Another example of the lack of clear separation between levels is that the effect of national wealth is mediated by religiosity, but it is unclear at which level the relation between religiosity and meaning in life exists: country or individual? Indirect effects are reported at the individual and the country level but these are estimated in separate models. This is unusual in multilevel modeling. For aggregated characteristics like religiosity, it is *essential* that these are only entered into the model when the individual-level variable is present at the same time (e.g., Snijders and Bosker, 2012, p. 17). If not, both levels are confounded. Thus, the indirect effects reported by OD at the national and individual-level are not in fact estimates of the indirect effect at either level, but in both analyses nation-level and individual-level effects are confounded. The best way to estimate effects at either level is to have *both* levels present in the same analysis.

As it turns out, such an analysis shows positive effects of religiosity at both levels. When nation-level religiosity (*B* = 0.222, *SE* = 0.036, *p* < 0.001) and individual-level religiosity (*B* = 0.049, *SE* = 0.005, *p* < 0.001) are together in the same model, both have a positive relation with meaning in life. This is similar to results when nation-level and individual-level religiosity were used in separate models (i.e., the results reported by OD), but in those models the individual and national level were confounded. It is important to understand that the effects could have differed between levels (as was the case for wealth). For example, in data from the 2005 to 2007 wave of the World Values Survey the item “How often do you think about the meaning and purpose of life?” is positively predicted by country religiosity but this positive relation decreases by 87% and is no longer significant once individual religiosity (“How important is God/religion in your life?”) is controlled for.

## Conclusion and recommendations

Drawing inferences about psychological processes using nation-level analyses presents a number of problems, some of which we have discussed here. Our additional analyses of HBW's and OD's data show that these are important issues that can change the substantive conclusions that can be drawn. We hope future work avoids these pitfalls and have a few recommendations for psychologists who are interested in using nation-level data.

First of all, researchers should identify the level at which their theory and hypotheses are situated. There needs to be a conceptual correspondence between theory and statistical analyses, otherwise one risks committing an ecological or atomistic fallacy. Multilevel modeling, when correctly applied, can partially help to avoid these mistakes (Hox, 2010; Snijders and Bosker, 2012). Our second recommendation is to be critical of the quality and reliability of the variables one uses in analyses. For nation-level analyses, the measurement equivalence between countries is an additional reliability issue one needs to take into account. Third, country-level data hardly ever satisfy the statistical assumption of independent sampling, because many country-level variables are clustered in regions. Such clustering can be partially corrected for by controlling for region in the analyses, but there are also other options such as phylogenetic analyses (Mace and Pagel, 1994), spatial modeling via the geographic information system (Chang, 2003), or modeling the autocorrelation (Dow, 2007).

## Conflict of interest statement

The authors declare that the research was conducted in the absence of any commercial or financial relationships that could be construed as a potential conflict of interest.
