# Supplementary material for: Mind the level: problems with two recent nation-level analyses in psychology
Source: Front Psychol. 2014 Sep 30;5:1110. doi: 10.3389/fpsyg.2014.01110 (PMC4179703; doi:10.3389/fpsyg.2014.01110)
Supplement: Supplementary file 1 [file DataSheet1.DOCX]

***Supplementary Material***

**Mind the level: Problems with two recent nation-level analyses in psychology**

**Toon Kuppens^1^*, Thomas V. Pollet^2^**

^1^Faculty of behavioural and social sciences, University of Groningen, Netherlands

^2^Faculty of psychology and education, VU University, Amsterdam, Netherlands

*** Correspondence:** Toon Kuppens, Department of social psychology, Faculty of behavioural and social sciences, University of Groningen, Grote Kruisstraat 2/1, 9712 TS Groningen, The Netherlands. t.kuppens@rug.nl

1. **Supplementary information on analysis of Hershfield, Bang and Weber (2014) data**

When controlling for region as we did with the data of HBW, one key question is how to divide countries into regions. As we stated in the main text, our particular division of countries into regions is merely based on our own assessment of geographical proximity and cultural and political similarities. Without any doubt, other researchers would have chosen slightly different divisions in region. We therefore wanted to check the robustness of our analyses by using alternative divisions into region. First, we used a different division into ten rather than twelve regions that are more in line with Murdock’s world regions (see http://en.wikipedia.org/wiki/Standard_cross-cultural_sample). Second, we also repeated the analysis using 19 “UN geographical regions for statistical use” (see http://unstats.un.org/unsd/methods/m49/m49regin.htm). Both results were similar to the results reported in the article: the effect of country age on EPI was not significant (*B* = .024 and .016, *p* = .14 and .30, respectively).

The size of the reduction in the effect of country age when adding ‘region’ to the model, seems to be related to the number of regions that are distinguished. The higher the number of regions that are distinguished, the smaller the effect of country age becomes. Indeed, when the region variable distinguishes only between the six continents, the effect of country age remains larger and is still marginally significant (*B* = .30, *p* = .054). However, six regions can of course not capture the variety of geographical, historical, and cultural similarities between countries.

In our discussions about his article and data, Dr. Hal Hershfield has suggested that our results might be due to multicollinearity issues. For the sake of completeness we note that our results also hold when region is used as a random rather than a fixed effect, which avoids collinearity between country age and region. Furthermore, the tolerance value for country age is never lower than .33, which suggests acceptable levels of collinearity. Finally, even if there was high multicollinearity between country age and region, this would only support our point about the need to control for region, rather than invalidate our analyses.

It is important to note that we have used GDP as a control variable in all models, in order to be consistent with HBW. In our opinion, it would have been better to control for GDP per capita. Given that GDP is a factor of the number of inhabitants, GDP per capita is a better measure of the relative wealth of countries.

Below are three tables with the divisions of countries into regions.

Table 1: Countries and regions (classification used in analyses reported in article)

| Northern and Western Europe | Austria, Belgium, Switzerland, Germany, Denmark, Finland, France, United Kingdom, Ireland, Iceland, Luxembourg, Netherlands, Norway, Sweden |
| --- | --- |
| Southern Europe | Albania, Bosnia and Herzegovina, Cyprus, Spain, Greece, Croatia, Italy, Macedonia, Malta, Portugal, Serbia, Slovenia |
| Central and Eastern Europe | Bulgaria, Belarus, Czech Republic, Estonia, Georgia, Hungary, Lithuania, Latvia, Republic of Moldova, Poland, Romania, Slovakia, Ukraine |
| Middle East | United Arab Emirates, Egypt, Iran, Iraq, Israel, Jordan, Kuwait, Lebanon, Oman, Qatar, Saudi Arabia, Syria, Turkey, Yemen |
| Caribbean | Cuba, Dominican Republic, Haiti, Jamaica, Trinidad and Tobago |
| Central America | Costa Rica, Guatemala, Honduras, Mexico, Nicaragua, Panama, El Salvador |
| South America | Argentina, Bolivia, Brazil, Chile, Colombia, Ecuador, Peru, Paraguay, Uruguay, Venezuela |
| North Africa | Algeria, Libya, Morocco, Tunisia |
| Africa | Angola, Benin, Botswana, Côte d’Ivoire, Cameroon, Democratic Republic of the Congo, Republic of the Congo, Eritrea, Ethiopia, Gabon, Ghana, Kenya, Mozambique, Namibia, Nigeria, Sudan, Senegal, Togo, Tanzania, South Africa, Zambia, Zimbabwe |
| Central and East Asia | Armenia, Azerbaijan, China, Japan, Kazakhstan, Kyrgyzstan, Mongolia, Nepal, Pakistan, Russian Federation, Tajikistan, Turkmenistan, Taiwan, Uzbekistan |
| South and Southeast Asia | Bangladesh, Brunei Darussalam, Indonesia, India, Cambodia, South Korea, Sri Lanka, Myanmar, Malaysia, Philippines, Singapore, Thailand, Vietnam |
| Anglo-Saxon | Australia, Canada, New Zealand, United States |

Table 2: Countries and regions (alternative classification, more based on Murdock)

| Northern and Western Europe | Austria, Belgium, Switzerland, Germany, Denmark, Finland, France, United Kingdom, Ireland, Iceland, Luxembourg, Netherlands, Norway, Sweden |
| --- | --- |
| Southern Europe | Albania, Bosnia and Herzegovina, Cyprus, Spain, Greece, Croatia, Italy, Macedonia, Malta, Portugal, Serbia, Slovenia |
| Central and Eastern Europe | Bulgaria, Belarus, Czech Republic, Estonia, Hungary, Lithuania, Latvia, Republic of Moldova, Poland, Romania, Russian Federation, Slovakia, Ukraine |
| Middle East and North Africa | United Arab Emirates, Armenia, Azerbaijan, Algeria, Egypt, Eritrea, Ethiopia, Georgia, Israel, Jordan, Kuwait, Lebanon, Libya, Morocco, Oman, Qatar, Saudi Arabia, Sudan, Syria, Tunisia, Turkey, Yemen |
| Africa | Angola, Benin, Botswana, Côte d’Ivoire, Cameroon, Democratic Republic of the Congo, Republic of the Congo, Gabon, Ghana, Kenya, Mozambique, Namibia, Nigeria, Senegal, Togo, Tanzania, South Africa, Zambia, Zimbabwe |
| North America | Canada, Mexico, United States |
| Caribbean and Central America | Costa Rica, Cuba, Dominican Republic, Guatemala, Honduras, Haiti, Jamaica, Nicaragua, Panama, El Salvador Trinidad and Tobago |
| South America | Argentina, Bolivia, Brazil, Chile, Colombia, Ecuador, Peru, Paraguay, Uruguay, Venezuela |
| Asia | Bangladesh, China, India, Iran, Iraq, Japan, Kazakhstan, Kyrgyzstan, Cambodia, South Korea, Sri Lanka, Myanmar, Mongolia, Malaysia, Nepal, Pakistan, Singapore, Thailand, Tajikistan, Turkmenistan, Taiwan, Uzbekistan, Vietnam |
| Insular Pacific | Australia, Brunei Darussalam, Indonesia, New Zealand, Philippines |

Table 3: UN geographical regions for statistical use

| Eastern Europe | Bulgaria, Belarus, Czech Republic, Hungary, Republic of Moldova, Poland, Romania, Russian Federation, Slovakia, Ukraine |
| --- | --- |
| Northern Europe | Denmark, Finland, United Kingdom, Ireland, Iceland, Norway, Sweden, Estonia, Lithuania, Latvia |
| Southern Europe | Albania, Bosnia and Herzegovina, Spain, Greece, Croatia, Italy, Macedonia, Malta, Portugal, Serbia, Slovenia |
| Western Europe | Austria, Belgium, Switzerland, Germany, France, Luxemburg, Netherlands |
| Northern America | Canada, United States |
| Caribbean | Cuba, Dominican Republic, Haiti, Jamaica, Trinidad and Tobago |
| Central America | Costa Rica, Guatemala, Honduras, Mexico, Nicaragua, Panama, El Salvador |
| South America | Argentina, Bolivia, Brazil, Chile, Colombia, Ecuador, Peru, Paraguay, Uruguay, Venezuela |
| Eastern Africa | Eritrea, Ethiopia, Kenya, Mozambique, Tanzania, Zambia, Zimbabwe |
| Middle Africa | Angola, Cameroon, Democratic Republic of the Congo, Republic of the Congo, Gabon |
| Northern Africa | Algeria, Egypt, Libya, Morocco, Sudan, Tunisia |
| Southern Africa | Botswana, Namibia, South Africa |
| Western Africa | Benin, Côte d’Ivoire, Ghana, Nigeria, Senegal, Togo |
| Central Asia | Kazakhstan, Kyrgyzstan, Tajikistan, Turkmenistan, Uzbekistan |
| Eastern Asia | China, Japan, Mongolia, South Korea, Taiwan |
| Southern Asia | Bangladesh, India, Iran, Sri Lanka, Nepal, Pakistan |
| South-Eastern Asia | Brunei Darussalam, Cambodia, Indonesia, Myanmar, Malaysia, Philippines, Singapore, Thailand, Vietnam |
| Western Asia | Armenia, Azerbaijan, Cyprus, United Arab Emirates, Georgia, Iraq, Israel, Jordan, Kuwait, Lebanon, Oman, Qatar, Saudi Arabia, Syria, Turkey, Yemen |
| Australia and New Zealand | Australia, New Zealand |
